# Vibration Alert to the Brain: Evoked and Induced MEG Responses to High-Frequency Vibrotactile Stimuli on the Index Finger of Dominant and Non-dominant Hand

**DOI:** 10.3389/fnhum.2020.576082

**Published:** 2020-11-05

**Authors:** Min-Young Kim, Hyukchan Kwon, Tae-Heon Yang, Kiwoong Kim

**Affiliations:** ^1^Quantum Technology Institute, Korea Research Institute of Standards and Science, Daejeon, South Korea; ^2^Department of Electronic Engineering, Korea National University of Transportation, Chungju-si, South Korea; ^3^Department of Medical Physics, University of Science and Technology, Daejeon, South Korea

**Keywords:** magnetoencephalography (MEG), vibrotactile stimulation, time-locked responses, alpha suppression, beta suppression

## Abstract

**Background:** In recent years, vibrotactile haptic feedback technology has been widely used for user interfaces in the mobile devices. Although functional neuroimaging studies have investigated human brain responses to different types of tactile inputs, the neural mechanisms underlying high-frequency vibrotactile perception are still relatively unknown. Our aim was to investigate neuromagnetic brain responses to high-frequency vibrotactile stimulation, using magnetoencephalography (MEG).

**Methods:** We measured 152-channel whole-head MEG in 30 healthy, right-handed volunteers (aged 20–28 years, 15 females). A total of 300 vibrotactile stimuli were presented at the tip of either the left index finger or the right index finger in two separate sessions. Sinusoidal vibrations at 150 Hz for 200 ms were generated with random inter-stimulus intervals between 1.6 and 2.4 s. Both time-locked analysis and time-frequency analysis were performed to identify peak responses and oscillatory modulations elicited by high-frequency vibrations. The significance of the evoked and induced responses for dominant and non-dominant hand stimulation conditions was statistically tested, respectively. The difference in responses between stimulation conditions was also statistically evaluated.

**Results:** Prominent peak responses were observed at 56 ms (M50) and at 100 ms (M100) for both stimulation conditions. The M50 response revealed clear dipolar field patterns in the contralateral side with significant cortical activations in the contralateral primary sensorimotor area, whereas the M100 response was not as prominent as the M50. Vibrotactile stimulation induced significant suppression of both alpha (8–12 Hz) and beta (20–30 Hz) band activity during the mid-latency period (0.2–0.4 s), primarily in sensorimotor areas contralateral to the stimulation side. In addition, a significant alpha enhancement effect in posterior regions was accompanied with alpha suppressions in sensorimotor regions. The alpha suppression was observed in a broader distribution of cortical areas for the non-dominant hand stimulation.

**Conclusion:** Our data demonstrate that high-frequency tactile vibrations, which is known to primarily activate Pacinian corpuscles, elicit somatosensory M50 and M100 responses in the evoked fields and induce modulations of alpha and beta band oscillations during mid-latency periods. Our study is also consistent with that the primary sensorimotor area is significantly involved in the processing of high-frequency vibrotactile information with contralateral dominance.

## 1. Introduction

The cutaneous sensory system provides information about the external environment by detecting tactile, thermal, and painful stimuli applied to the skin through mechanoreceptors, thermoreceptors, and nociceptors, respectively (Birder and Perl, 1994; McGlone and Reilly, 2010). Tactile mechanoreceptors respond to mechanical distortion of the skin, and there are four main types of mechanoreceptors in glabrous skin. Pacinian corpuscles are primarily responsible for detecting high frequency vibrations (40–500 Hz), Meissner corpuscles for low frequency flutters (2–40 Hz), Merkel's disks for light touch or sustained pressure, and Ruffini endings are sensitive to skin stretch (Johansson and Vallbo, 1979, 1983; Bolanowski et al., 1988; Johnson, 2001). In contrast to traditional views of specialized functional roles for each type of mechanoreceptors, recent findings have indicated that inputs from multiple mechanoreceptor types are integrated in the cortex for tactile information processing (Bensmaia, 2008; Tommerdahl et al., 2010; Carter et al., 2014; Saal and Bensmaia, 2014; Kuroki et al., 2017). Signals from different mechanoreceptors contribute to the characterization of tactile sensation that include vibration, shape, motion, grip control, and texture (Saal and Bensmaia, 2014). Due to the complex nature of tactile signals, clearly defining the physical properties of stimuli and producing natural tactile sensations have been challenging issues in this field of research.

In recent years, with the ubiquity of touchscreen interfaces, vibrotactile feedback technology has received increased attention as an effective communication channel for enhanced user interaction using touchscreen devices (Hoggan et al., 2008; Jones and Sarter, 2008; Choi and Kuchenbecker, 2013). There has been active research on the fundamentals of vibrotactile perception through both psychophysical and neurophysiological measurements, with most studies focusing on the threshold for the detection of vibrations (Johansson et al., 1982; Verrillo, 1985; Bolanowski et al., 1988; Gescheider et al., 2002; Morioka and Griffin, 2005; Ryu et al., 2010; Jones and Tan, 2013). Human skin is found to be most sensitive to vibrations at frequencies between 150 and 300 Hz, where the Pacinian channels are predominantly activated (Jones and Sarter, 2008), and various types of actuator techniques have been introduced to stimulate vibrotactile sensation using solenoids, voice coils, rotary DC motors, piezoelectric actuators, electroactive polymer actuators, pneumatic actuators, etc. (Choi and Kuchenbecker, 2013).

Although functional neuroimaging studies have shown that brain responses to high-frequency vibrations are different from responses to low-frequency flutter (Hämäläinen et al., 1990; Hashimoto et al., 1998; Harrington and Downs, 2001; Tommerdahl et al., 2010; Chung et al., 2013), the neural mechanisms underlying high frequency vibrotactile perception are still relatively unknown. Since neuronal information processing for vibrotactile perception occurs at a millisecond timescale (Jousmäki, 2000; Mackevicius et al., 2012), non-invasive neuroimaging methods, such as electroencephalography (EEG) or magnetoencephalography (MEG) can play important roles in assessing vibrotactile information processing due to their high temporal resolution (Kekoni et al., 1997; Hashimoto et al., 1998; Jousmäki and Hari, 1999; Tobimatsu et al., 1999; Tuunanen et al., 2003; Nangini et al., 2006). While EEG signals are distorted by a multi-layered head structure with inhomogeneous electric conductivities, MEG signals are much less influenced by different tissue properties because they have constant magnetic permeability, thereby giving more accurate source estimation results (da Silva, 2013; Baillet, 2017).

Based on its higher sensitivity to tangential sources, MEG has been actively used to examine human somatosensory cortical activity, distributed mainly along the central and lateral sulci of the brain (Hari and Forss, 1999; Kakigi et al., 2000; Nevalainen et al., 2014). Most previous MEG studies investigated tactile responses to light touch or flutters that were generated by using air-puffs (Forss et al., 1994; Rossini et al., 1996), pneumatic stimulators (Yang et al., 1993; Mertens and Lütkenhöner, 2000; Hoechstetter et al., 2001; Nangini et al., 2006), or brushes (Cheyne et al., 2003; Jousmäki et al., 2007). Due to difficulties in producing natural high-frequency vibrations in a precise manner without electromagnetic artifacts, there have been only a few studies examining neuromagnetic brain responses to high-frequency vibrotactile stimuli generated by a piezoelectric stimulator (Hashimoto et al., 1998; Iguchi et al., 2007; Onishi et al., 2010) or a loudspeaker system (Jousmäki and Hari, 1999) with limitations that include humming sounds and temporal amplitude variations. In order to reveal the contributions of different mechanoreceptors to complex aspects of tactile sensations, more natural stimulation paradigms under well-controlled conditions are essential.

In an attempt to cope with the increasing demands for an objective evaluation of high frequency vibrotactile perception, we investigated the neuromagnetic responses to high frequency vibrotactile stimulation, using a custom-built, MEG-compatible vibrotactile device. In our earlier report (Kim et al., 2019), we successfully demonstrated the operation of our new device in an MEG environment without producing artifacts and showed prominent peak responses evoked by vibrotactile stimuli applied to the right index finger. In the present work, we expanded our analysis to include MEG data obtained by stimulating the left index finger or right index finger in two separate sessions. We employed time-lock analysis of the MEG data to identify topological distributions of peak responses in sensor space and then performed source analysis to estimate cortical activation patterns at the peaks. The differences between stimulus conditions, both in topographic sensor maps and in source activation patterns, were statistically evaluated for significance. Additionally, we also applied time-frequency analysis to examine MEG oscillatory responses induced by high-frequency vibrotactile input. In particular, we investigated the modulations in alpha band power (8–12 Hz) and beta band power (20–30 Hz) during vibrotactile tasks. Neuromagnetic somatosensory responses, both evoked responses and cortical oscillations, have been intensively investigated for clinical applications (Hari and Forss, 1999; Kakigi et al., 2000; Cheyne, 2013). Our results of using a polymer-based tactile actuator for studying neuromagnetic responses to high-frequency vibrations may provide valuable information for the application of vibrotactile haptic feedback technology, which is now ubiquitous in daily lives, in basic and clinical research.

## 2. Materials and Methods

### 2.1. Participants

Thirty healthy young adults (aged 20–28 years, 15 females) were recruited from local universities. Inclusion criteria were normal or corrected-to-normal vision, no personal history of neurological or psychiatric disorders, and right-hand dominance assessed by the revised Edinburgh Handedness Inventory (Oldfield, 1971). Written informed consent was obtained from all participants. The MEG data were recorded at the Korea Research Institute of Standards and Science (KRISS) with approval by the Institutional Review Board on Human Subjects Research and Ethics Committee (KRISS-IRB-2016-07). All experimental procedures were performed in accordance with the Declaration of Helsinki. For each participant, the locations of four head position indicator coils in relation to three fiducials (nasion, and the right and left pre-auriculars) were digitized using a 3D digitizer (ISOTRACK II, Polhemus, Colchester, VT, USA), with ~65 additional points from the scalp to represent individual head shapes prior to the MEG recordings. We measured the locations of the head position indicator coils before and after each recording session. The data from one participant was excluded from the analysis because the exact head position within the MEG helmet was not available due to the movement of head position indicator coils on the head surface during the recordings.

The stimulus paradigm, experimental designs, and data acquisition have been described in detail in Kim et al. (2019), which reported preliminary results of evoked responses to vibrotactile stimulation at the tip of the right index finger only. In the present work, data collected from the same group of participants for stimulation at the tip of the left index finger were added with expanded analyses.

### 2.2. Stimuli and Experimental Paradigm

**Figure 1:**
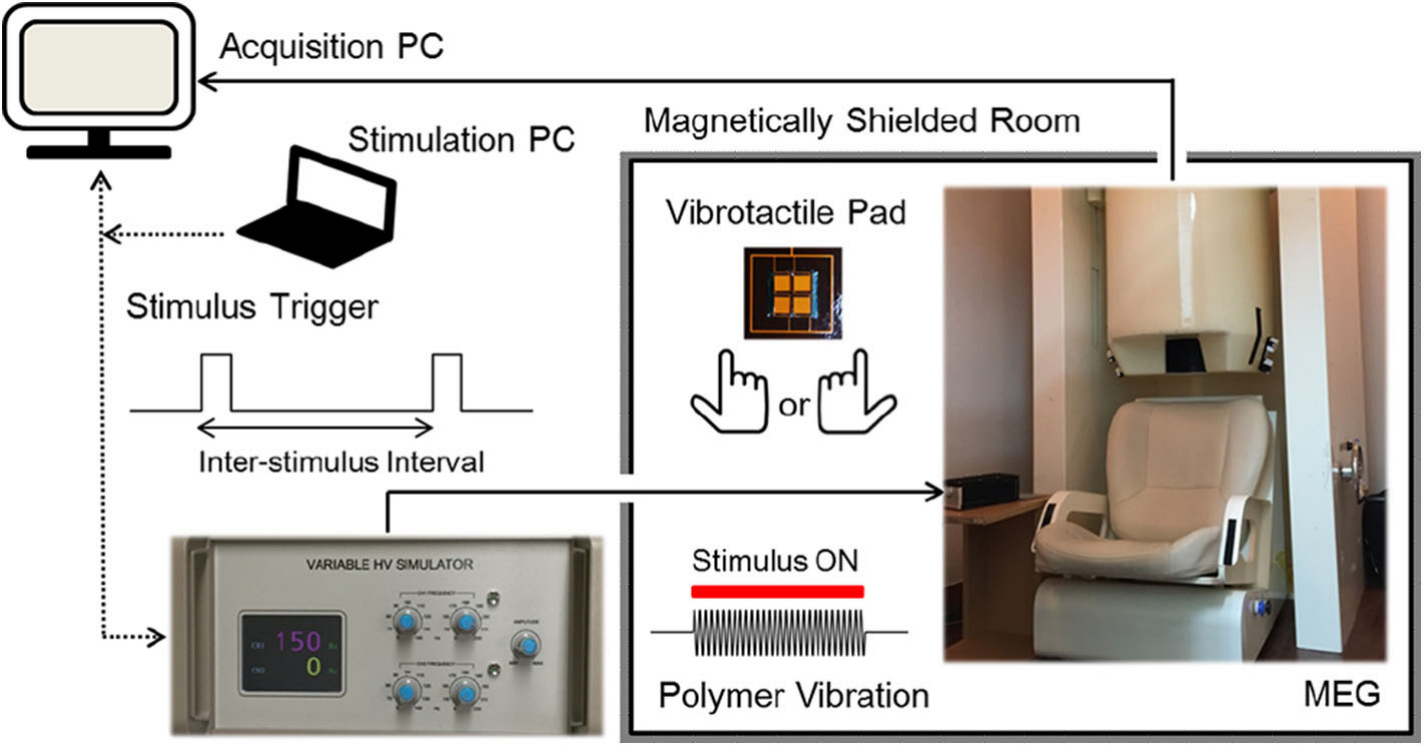
Experimental setup. Participants were comfortably seated on a chair in a magnetically shielded room with their heads placed inside a helmet of an MEG dewar. Sinusoidal vibrations were presented to the tip of the left index finger or the right index finger in two separate sessions, in a counterbalanced order. A total of 300 stimuli were applied at 150 Hz for a 200 ms duration with random inter-stimulus intervals between 1.6 and 2.4 s [see Kim et al. (2019) for details].

Vibrotactile stimulation was delivered to the tip of the index finger using an MEG-compatible polymer based tactile actuator developed in our laboratory (see Kim et al. (2019) for details). Sinusoidal vibrations of the actuator were produced by using a variable, high voltage simulator which controlled stimulus frequency, amplitude, and duration with external triggers from a stimulation PC (Figure 1). Participants were comfortably seated on a chair in a magnetically shielded room with their heads placed inside a helmet of an MEG dewar. They were asked to relax and to not move their heads during the MEG recordings. They placed their index finger of either the right hand or the left hand gently on a vibrotactile pad (20 × 20 mm in size), without pressing on it. The right index finger and the left index finger were stimulated in two separate sessions for about 10 min each, where the order of the stimulated hand was counterbalanced across participants. In each session, a total of 300 vibrotactile stimuli at a frequency of 150 Hz for a 200 ms duration were applied to the tip of the index finger with random inter-stimulus intervals (ISI) between 1.6 and 2.4 s. Before each session, the participants were familiarized with the vibrotactile stimuli, and adjusted the position of their index finger on the tactile pad for optimal sensitivity. We used a fixed vibration amplitude at the maximum level of the stimulation controller and confirmed that all participants were able to clearly detect the tactile vibrations prior to the actual recordings. Participants were instructed to gaze at fixation mark in front of them to reduce ocular activity and to pay attention to the vibratory stimuli presented at the fingertip. All MEG experiments were conducted in a quiet environment with no report of hearing any auditory sound during the stimulations.

### 2.3. MEG Data Recording and Pre-processing

The neuromagnetic responses were measured by using a helmet-shaped MEG system with 152 first-order axial gradiometers [KRISSMEG, Daejeon, South Korea (Lee et al., 2009; Kim et al., 2013)]. MEG signals were recorded with a hardware low-pass filter at 234 Hz and digitized at a sample rate of 1,024 samples/s. We performed preprocessing and sensor space analysis using the FieldTrip toolbox (Oostenveld et al., 2011) and source space analysis using Brainstorm toolbox (Tadel et al., 2011). Both FieldTrip and Brainstorm are open source toolboxes that run in MATLAB (MathWorks, Natick, Massachusetts, USA) for MEG/EEG analysis. Continuous raw data were imported with a band-pass filter of 0.1–511 Hz and decomposed with the second-order blind identification (SOBI) algorithm implemented in FieldTrip. Independent components corresponding to ocular, respiratory, cardiac artifacts, and power line noises, were visually identified and removed to reconstruct clean MEG data. The clean signals were segmented from −1 to 1.6 s with respect to stimulus onset, and trials with large variances, due to mental fatigue or muscle activities, were rejected from further analysis. On average, 291 ± 5 trials out of 300 for both stimulus conditions were identified as good trials.

### 2.4. Event Related Fields

The clean data were low-pass filtered at 50 Hz, epoched with respect to each stimulus presentation including the time window of interest (−0.5–1.5 s) with a 0.5 s buffer, and were baseline corrected by removing the mean values of each sensor in a time interval of −0.4 to −0.2 s from each sample point. Individual event-related fields (ERFs) were obtained by producing trial averages for each stimulus condition. In sensor space, we computed the average MEG sensor positions across all participants for both stimulus conditions, and interpolated the ERFs of each participant to the average sensor positions using the “ft_megrealign” function of the FieldTrip toolbox. Grand-average ERFs (gERFs) were computed by averaging the realigned ERFs across 29 participants for each stimulus condition, and the global field power (GFP), corresponding to the spatial standard deviation across all sensors, was calculated to estimate the scalp field strength for the gERFs at each time point.

Prior to a comparison between MEG responses to the left index finger stimulation and responses to the right index finger stimulation, the presence of consistent topography across all participants was statistically evaluated independently for each stimulus condition using a Topographical Consistency Test (TCT) (Koenig and Melie-García, 2010) implemented in a MATLAB-based program RAGU (Randomization Graphical User Interface) (Koenig et al., 2011). A Topographic Analysis of Variance (TANOVA), which is also implemented in the RAGU software, was conducted to compare topographic field differences across all sensors and time points between two stimulus conditions. Five thousand randomizations were performed with an alpha level of 0.05, and global duration statistics were calculated to control for multiple comparisons in time, where the duration of significant effects in real data needs to be longer than 95% of the significant periods (continuous periods with *p* < 0.05) in the randomized data.

Stimulation-related changes in topographic distributions of the grand averaged waveforms at the GFP peaks were statistically examined by comparing the mean values of the gERFs during an active time period for the corresponding peak responses with the mean values of the gERFs during a baseline time period (between −340 to −320 ms before onset). The mean values at peaks were calculated for 10 ms intervals around the peak at *t* = *t*_*peak*_ ([*t*_*peak*_ - 10 ms, *t*_*peak*_ + 10 ms]). For each stimulus condition, the mean primary peak responses, as well as the mean secondary peak responses, were compared with the mean baseline responses using a non-parametric cluster-based permutation test (Maris and Oostenveld, 2007) implemented in the Fieldtrip toolbox (Oostenveld et al., 2011). In addition, differences in the mean peak responses, as well as the differences in the mean baseline responses, between left and right index finger stimulation, were evaluated for both the primary and the secondary peak responses, using the same permutation tests. A paired sample *t*-test was used to assess significant differences between two experimental conditions at sample level with a critical alpha of 0.05. The sum of the *t*-values within every cluster was used as a cluster-level statistic, and the distribution of the maximum values of the summed *t*-values was obtained by 1,000 random permutation runs. Clusters with an observed cluster-level statistic falling in the highest or lowest 2.5% were considered to exhibit significant differences between the two conditions.

### 2.5. Source Analysis

Cortical source activations arising from the vibrotactile stimulations were estimated for each participant by applying the weighted minimum norm estimate method (wMNE) (Hämäläinen and Ilmoniemi, 1994; Lin et al., 2006) implemented in the Brainstorm toolbox (Tadel et al., 2011). We generated a pseudo-individual anatomy for each participant by deforming the ICBM152 template anatomy using the digitized head points of each individual. A head model was computed using the overlapping spheres method (Huang et al., 1999) for each stimulus condition with the original sensor position of the recorded data, and the noise covariance was estimated from the baseline period (−0.4 to −0.2 s) of all accepted trials. Current density values were computed for unconstrained orientations, i.e., three orientations at each vertex of the cortical surface, with default parameter settings for regularization and source depth weighting in the Brainstorm software. The current density values were averaged across trials for each participant and stimulus condition.

For group analyses, we calculated the norm of the trial averaged current density values at each vertex point and applied z-score transformation to each cortical source trace using its mean and standard deviation over the baseline period (−0.4 to −0.2 s) (Tadel et al., 2019). Source activations at the GFP peaks identified from the sensor space analysis were estimated by averaging the mean z-scores over the active period ([*t*_*peak*_ - 10 ms, *t*_*peak*_ + 10 ms]) across all participants for each stimulus condition. Significant differences in the cortical activation between left and right index finger stimulations were evaluated by a two-tailed permutation paired *t*-test (10,000 randomizations, α = 0.01, FDR corrected) implemented in Brainstorm.

### 2.6. Spectral Analysis

For the sensor level frequency analysis, the realigned trial data for axial gradiometer sensors were planar transformed using the “ft_megplanar” function from the FieldTrip toolbox, and power spectra computed separately for the horizontal and the vertical planar gradiometers were combined at each sensor location using the “ft_combineplanar” function. Oscillatory powers were calculated for each trial using Morlet wavelets (with seven cycles), for frequencies between 5 and 50 Hz over a time window from −0.5 to 1.5 s in steps of 10 ms. The spectral powers were averaged across trials for each stimulus condition, and were dB normalized with respect to the average power during the baseline period (−0.4 to −0.2 s) separately for every frequency.

The vibrotactile stimulation induced oscillatory powers were grand averaged in the sensor space across all participants for each stimulus condition. Significant differences in spatio-temporal patterns of the spectral powers between left and right stimulus conditions were evaluated by performing a cluster-based permutation test to the 3D-(sensor, frequency, time) power spectra (1,000 permutations, a significance level of 0.05) with a two-sided paired sample *t*-test as a sample statistic for a cluster-defining threshold of *p* < 0.05 (uncorrected). Based on the results of the significance test, we investigated stimulation induced modulations in alpha band power (8–12 Hz) and in beta band power (20–30 Hz) in subsequent analyses.

Temporal development of the alpha power distribution in the sensor space was visualized using the averaged power in a frequency range of 8–12 Hz for a 0.2 s time window advanced from −0.4 to 1.0 s in steps of 0.2 s without overlapping. The difference in alpha band activity between left and right stimulus conditions was plotted together with alpha band activity for the respective stimulus condition. The same procedure was applied to show the temporal development of the beta power distribution in a frequency range of 20–30 Hz. In particular, we evaluated the statistical significance of oscillatory power changes in an active period between 0.2 and 0.4 s after stimulus relative to the baseline period, using a cluster-based permutation test (1,000 permutations, *p* < 0.05 with a paired *t*-test for each sample and a significance level of 0.05), for alpha band and beta band, respectively. Differences in the oscillatory powers between left and right stimulus conditions, both during the active period and during the baseline period, were also statistically tested by repeating the cluster-based permutation approach.

Neuronal sources of alpha and beta oscillations were reconstructed by applying a Hilbert transform to the single trial source time series estimated with the wMNE method in the Brainstorm toolbox, as described in the previous section (“Source Analysis”). We filtered the three orthogonal signals at each vertex of the cortical surface in alpha (8–12 Hz) and beta (20–30 Hz) frequency bands, computed the Hilbert transform of the filtered signals, and then summed the squared magnitudes from three directions to obtain power time series for each trial at each vertex. Spectral power was averaged across all accepted trials for each time point and was dB transformed relative to the mean baseline power between −0.4 and −0.2 s at each vertex, for each frequency band and stimulus condition. As in the sensor space analysis, we compared the oscillatory source power changes in an active period between 0.2 and 0.4 s relative to baseline activity using a permutation paired *t*-test (10,000 randomizations, α = 0.01, FDR corrected) for each frequency band and stimulus condition. Statistically significant differences in the oscillatory source powers between left and right stimulus conditions during the active period (0.2–0.4 s) were assessed by repeating the permutation paired *t*-test (10,000 randomizations, α = 0.01, FDR corrected), for each frequency band.

## 3. Results

### 3.1. Time-Locked Response to Vibrotactile Stimulations

**Figure 2:**
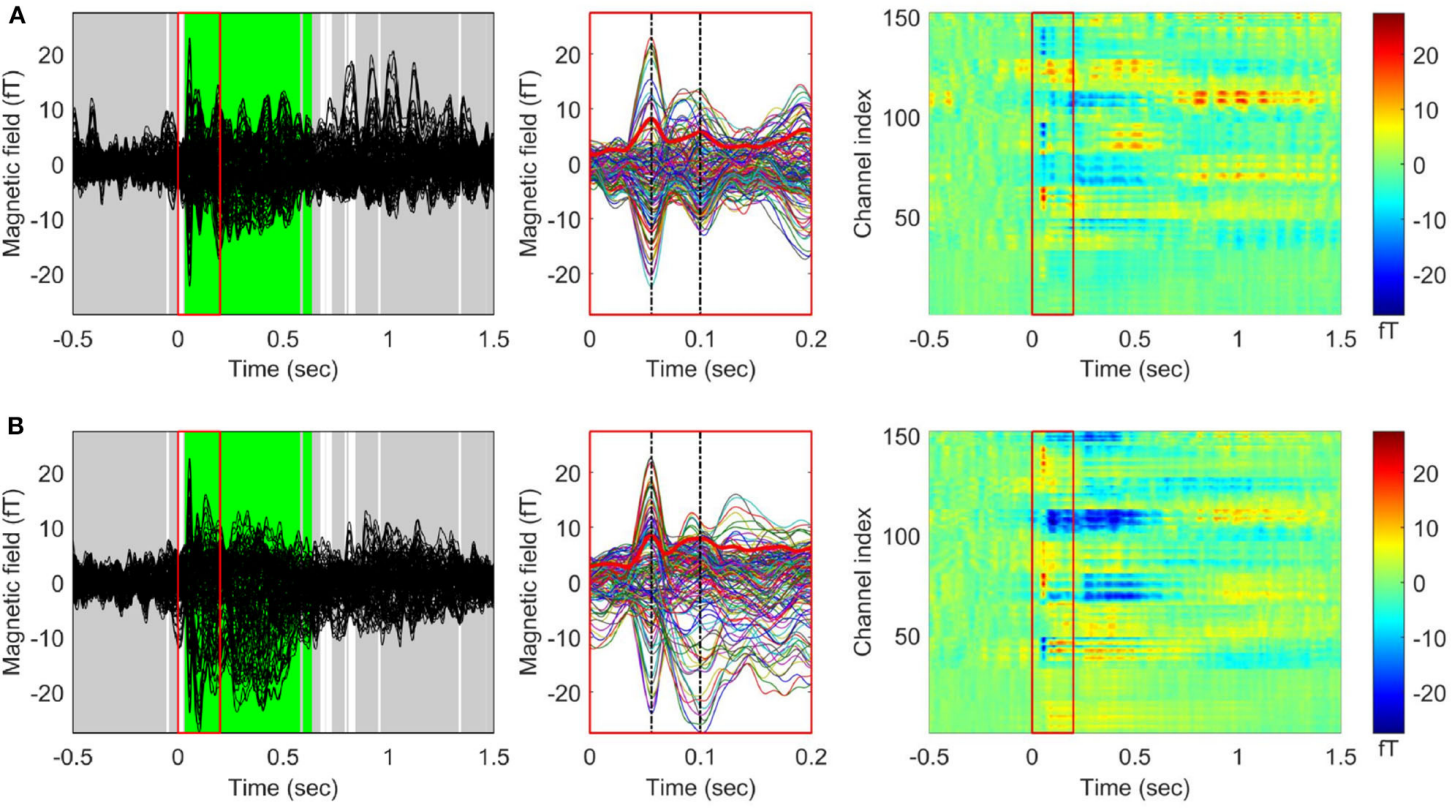
Grand averaged event related fields (ERF) in response to vibrotactile stimulation. Butterfly plots (left panel) and 2D color plots (right panel) of the ERF waveforms averaged across 29 participants **(A)** for the left index finger stimulation condition and **(B)** for the right index finger stimulation condition. The waveforms between 0 and 0.2 s (indicated by red boxes in the butterfly plots and 2D color plots) were enlarged in the middle panel. The thick red waveforms indicate the global field power (GFP) for each stimulation condition, and the dash-dotted vertical lines (black) were drawn at the first (M50) and the second (M100) peaks of the GFP waveforms. In the 2D color plots, y-axis indicates MEG channels and the color represents the value of magnetic fields. The results of applying topographic ANOVA (TANOVA) for the comparison between the grand averaged ERFs of each condition are displayed in the butterfly plots (5,000 randomizations). The time points with a *p*-value above the threshold (*p* > 0.05) are marked in gray while the time points with a *p*-value below the threshold (*p* < 0.05) are marked in white. The time periods satisfying the duration criterion are shown in green [see also Kim et al. (2019) for other representation of ERFs for the right index finger stimulation condition].

When the TCT was applied to the gERFs of each stimulus condition, we found a consistent topography across all participants beginning from the stimulus onset (approximately within 0–1.35 s except during a few brief periods, Supplementary Figure S1) for both conditions. The TANOVA results (Figure 2) showed that a significant difference between left and right stimulation was present from 33 to 580 ms, and from 595 to 637 ms. The global duration control statistic was 33 ms. The GFP waveforms of both stimulus conditions simultaneously reached their first peak at 56 ms (*t*_*p*1_, M50) and the second peak at 100 ms (*t*_*p*2_, M100).

**Figure 3:**
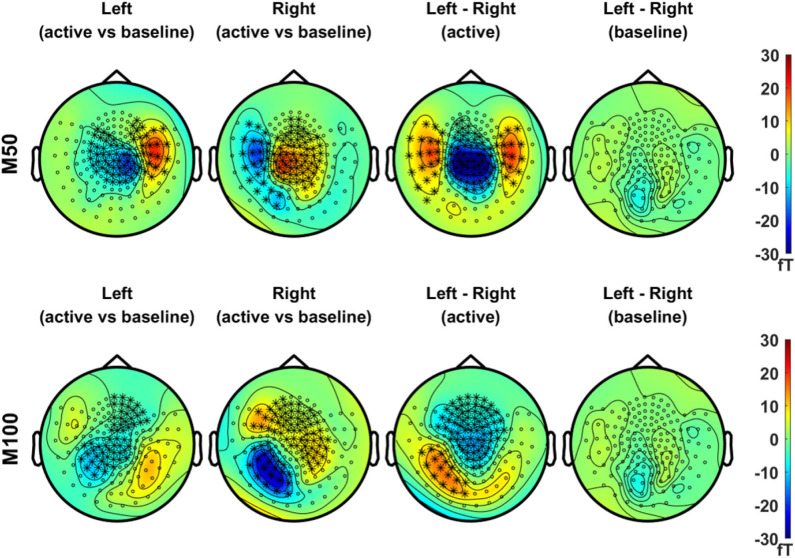
Topographical maps of peak MEG responses to vibrotactile stimulation. In the upper panel, field distributions of the grand averaged M50 responses (mean values of ERFs over an active period between 46 and 66 ms) after left (first column) and right (second column) index finger stimulation were statistically compared with their respective baseline level, separately (*N* = 29, cluster-based permutation test, 1,000 runs, cluster-defining threshold *p* < 0.05, α = 0.05). In addition, the difference in M50 between left and right stimulation conditions, as well as the difference in baseline activity between conditions, was statistically tested for significance (*N* = 29, cluster-based permutation test, 1,000 runs, cluster-defining threshold *p* < 0.05, α = 0.05). Channels associated with significant clusters are indicated by asterisks. In the lower panel, the same analyses were applied as the upper panel but with M100 responses (mean values of ERFs over an active period between 90 and 110 ms). All plots are obtained for axial gradiometers.

In Figure 3, we compared the magnetic field distributions of the first (M50, in the upper panel) and second (M100, in the lower panel) peak responses in the left and right stimulation conditions. The topographical maps of the grand-averaged M50 in both conditions showed significant sensor clusters with clear dipolar field patterns in the contralateral side. The field distributions of M100, on the other hand, showed bilateral responses for both conditions. Although the significant clusters for the M100 response to the respective stimulation condition, i.e., left or right, did not clearly reveal the dipolar patterns in each hemisphere, dipole-like responses became more discernible during the temporal development of the topographic maps illustrated in Supplementary Figures S2, S3.

**Figure 4:**
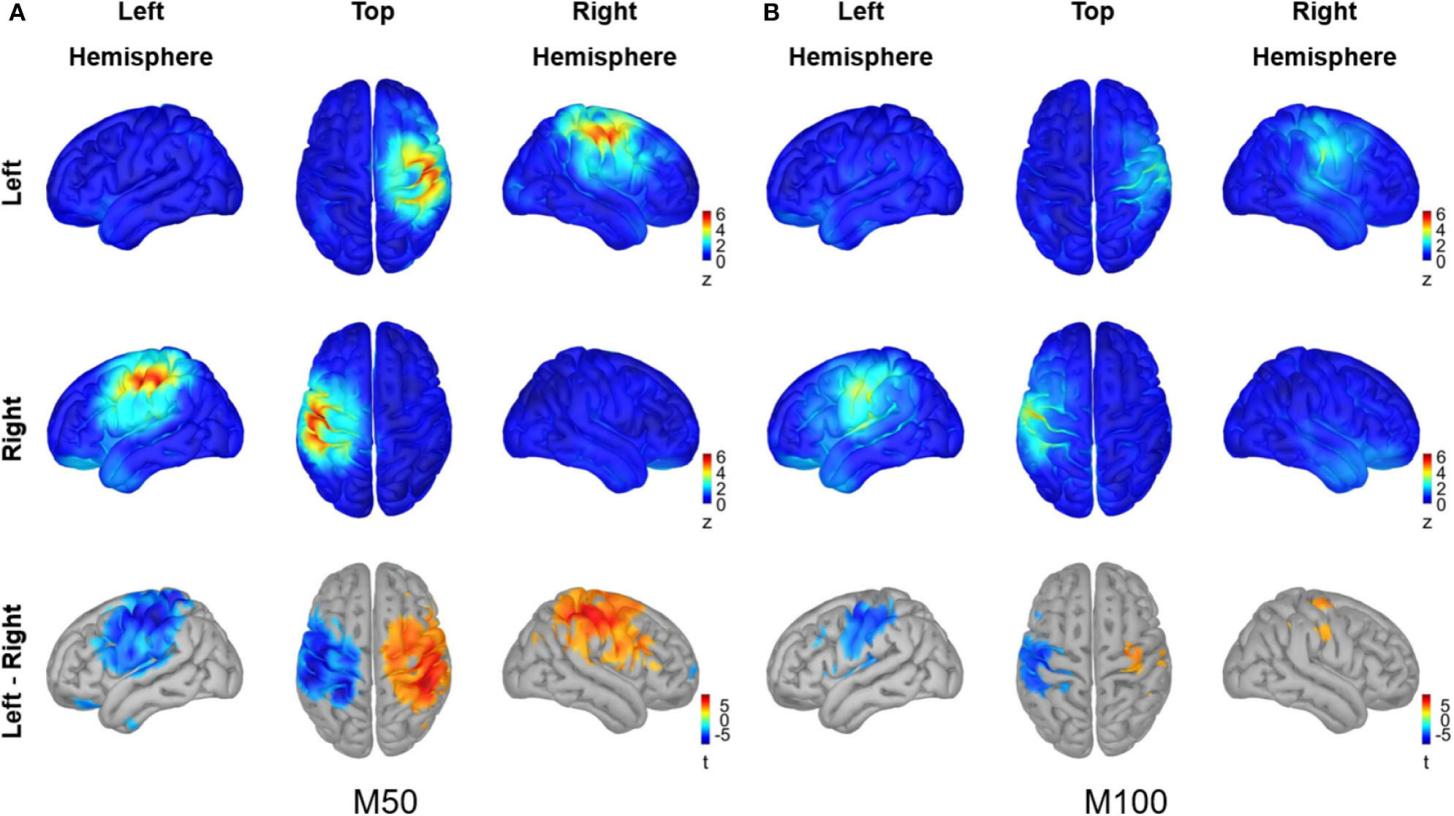
Source analysis of peak MEG responses to vibrotactile stimulation. The grand averaged source activity **(A)** for M50 responses and **(B)** for M100 responses are illustrated. The average z-scores with a 10 ms interval around each peak, i.e., 46–66 ms for M50 and 90–110 ms for M100, are plotted for the left index finger stimulation condition (top panel) and for the right index finger stimulation condition (middle panel), respectively. In the lower panel, the statistical t-maps of areas showing a significant difference between left and right stimulation conditions are plotted, with the red color indicating greater activity for the left index finger stimulation compared with the left index finger stimulation (permutation paired *t*-test, 10,000 randomizations, α = 0.01, FDR corrected). For each panel, views from the left hemisphere, top, and the right hemisphere are presented simultaneously.

The cortical sources for M50 and M100 responses in Figure 4 were visualized using average z values within a 10 ms interval around each peak (i.e., 46–66 ms for M50, 90–110 ms for M100), and the activity difference between conditions was statistically tested for each peak (permutation paired *t*-test, 10,000 randomizations, α = 0.01, FDR corrected). The M50 response most strongly occurred contralaterally to the stimulated hand over the primary sensorimotor (SMI) area. A significant difference between conditions for the M50 response was observed in the SMI area of each hemisphere. The M100 responses for both conditions, on the other hand, were not as prominent as the M50 responses and showed significant differences between conditions over the SMI cortex of the left hemisphere, driven by stronger activity in contralateral M100 for right index finger stimulation compared to left index finger stimulation.

### 3.2. Induced Response to Vibrotactile Stimulations

**Figure 5:**
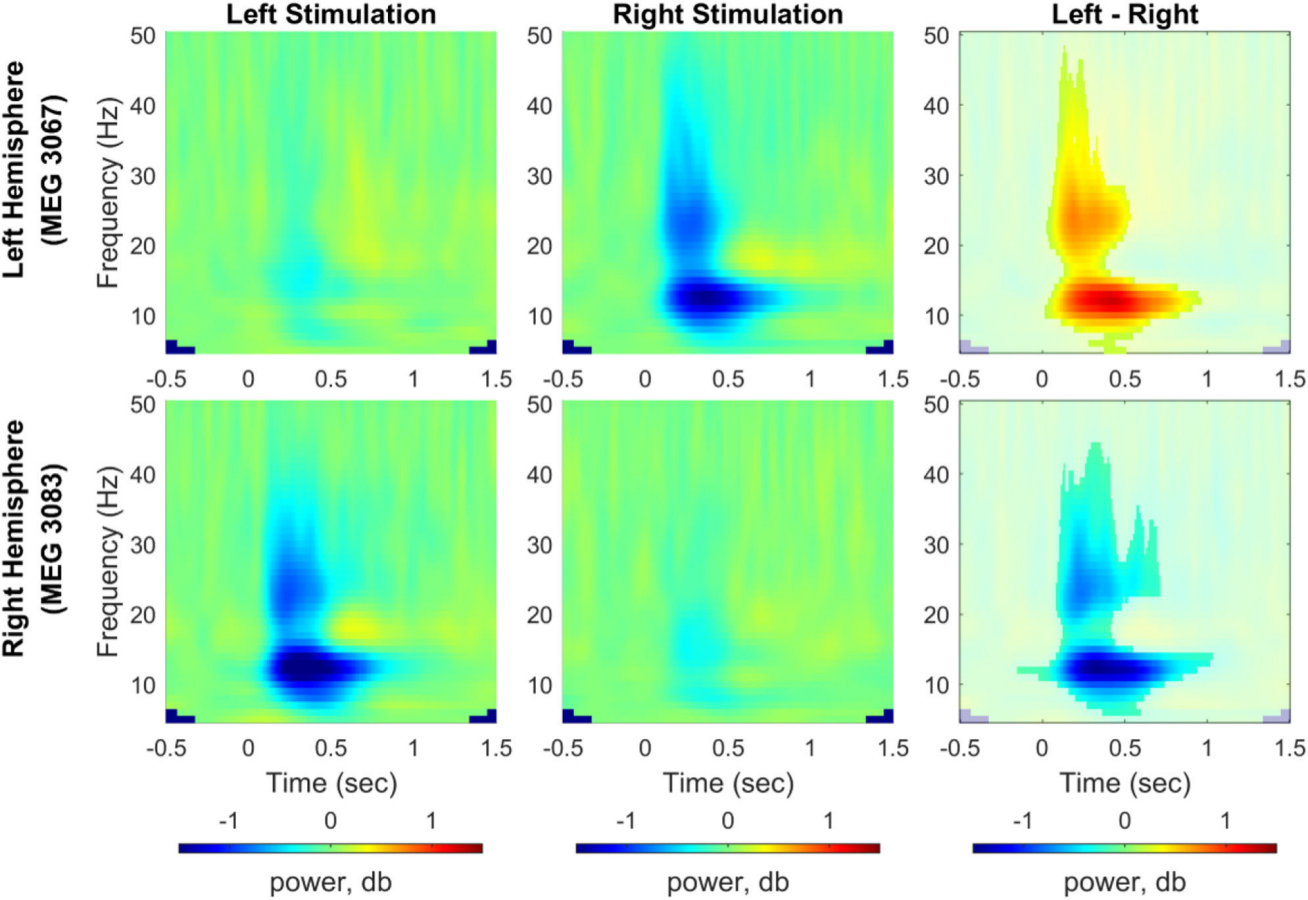
Time-frequency representation of spectral power change induced by vibrotactile stimulation. Grand averaged spectral power change relative to baseline obtained from representative channels from each hemisphere (MEG 3067 for the left hemisphere and MEG 3083 for the right hemisphere) was plotted for the left index finger stimulation condition (left panel) and for the right index finger stimulation condition (middle panel), respectively. In the right panel, power differences between left and right stimulation conditions associated with a significant cluster are shown non-blurred (*N* = 29, cluster-based permutation test, 1,000 runs, a significance level of 0.05). Blue color indicates power suppression relative to the baseline for each stimulation condition in the left and center panels, whereas it indicates lower spectral power for the left index finger stimulation compared with the right index finger stimulation (see Supplementary Figures S4–S7 for results from all MEG channels).

For induced responses, grand averages of spectral powers were calculated at each sensor across all participants after dB normalization of powers relative to the baseline activity. We compared induced responses between the left and right index finger stimulations using a cluster-based permutation test (1,000 permutations, a significance level of 0.05). Figure 5 shows the time-frequency representation of the grand averaged spectral powers for each stimulation condition (5–50 Hz, −0.5–1.5 s) and power differences between conditions from two representative channels, i.e., MEG 3067 from the left hemisphere and MEG 3083 from the right hemisphere (see Supplementary Figures S4–S7 for spectral powers from all channels). We observed a clear predominance of contralateral responses in the spectral powers for both stimulation conditions. In particular, power in the alpha (8–12 Hz) and beta (20–30 Hz) bands was significantly suppressed in the channels contralateral to the stimulated side.

**Figure 6:**
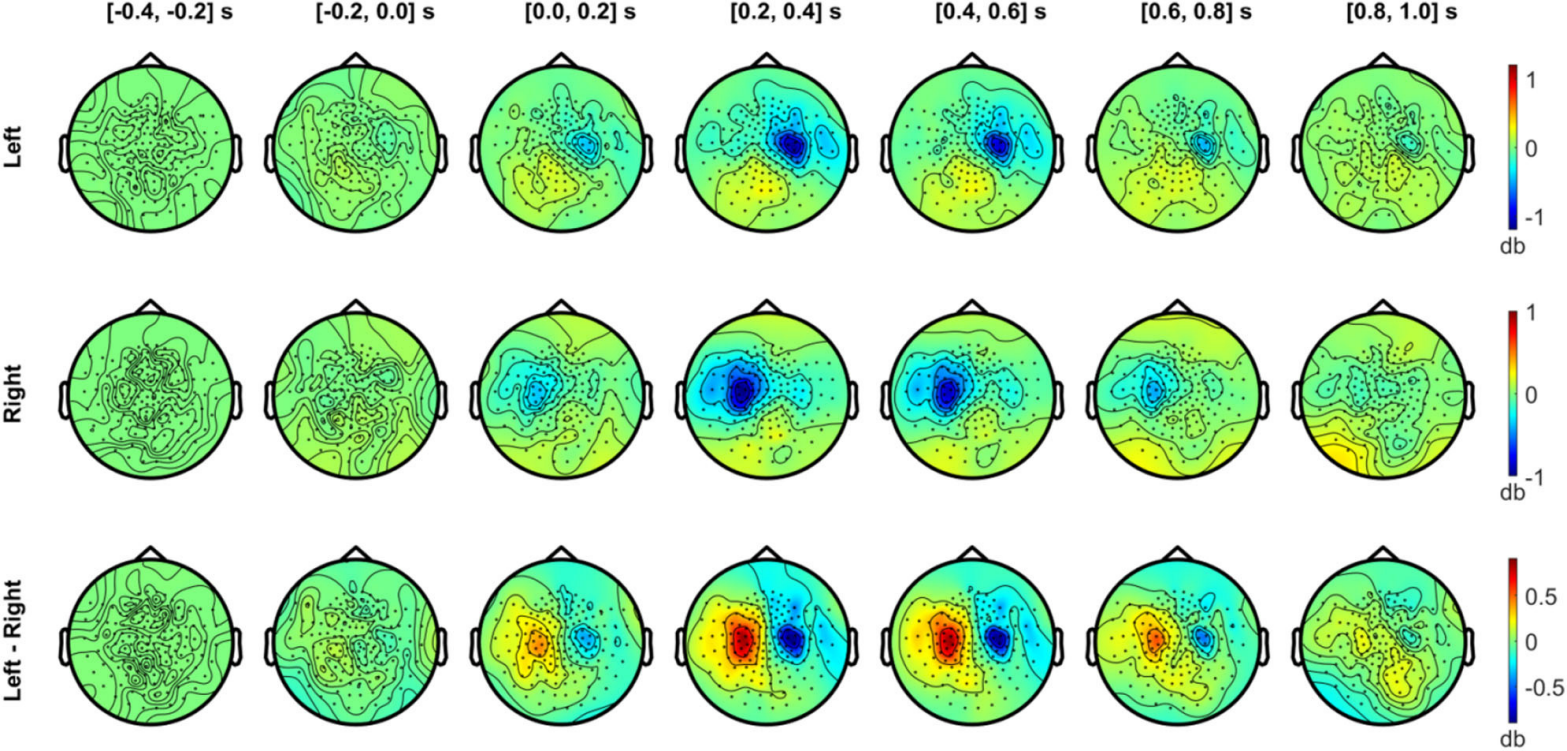
Temporal development of topographical power distributions in the alpha band (8–12 Hz) induced by vibrotactile stimulation. The topography of spectral power change in the alpha band (8–12 Hz) relative to the baseline is plotted for every 0.2 s, between −0.4 to 1.0 s with respect to the stimulus onset, both for the left index finger stimulation (top panel) and for the right index finger stimulation (middle panel). For both conditions, a contralateral alpha suppression (blue colors) effect is clearly observed in sensors overlying the sensorimotor regions. In the bottom panel, the difference in the alpha power between left and right stimuli is plotted. Blue areas indicate a lower alpha activity in the left index finger stimulation compared with the right index finger stimulation, and vice versa for red areas. All plots are obtained for planar gradiometers.

**Figure 7:**
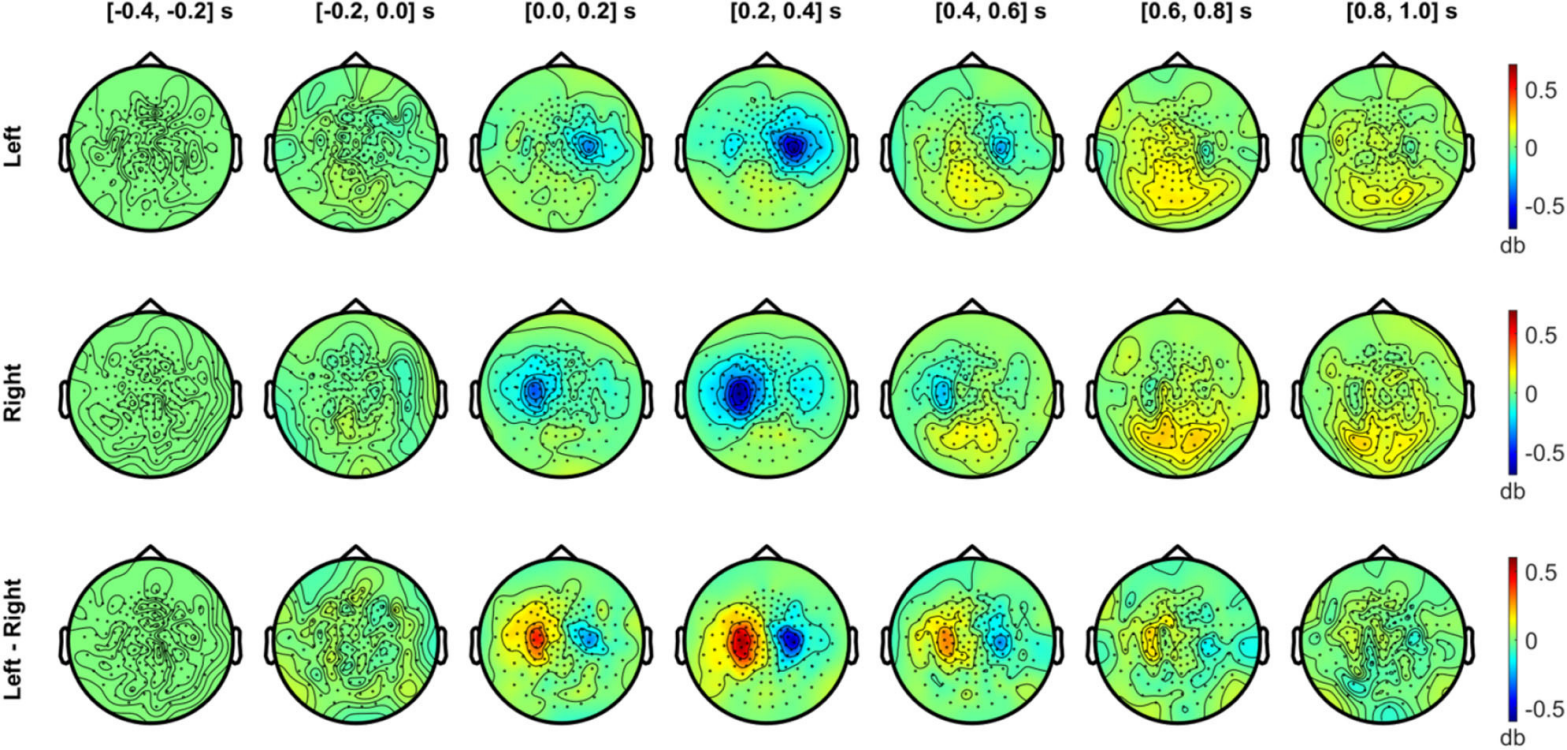
Temporal development of topographical power distributions in the beta band (20–30 Hz) induced by vibrotactile stimulation. The same format as Figure 6 is used but for beta band (20–30 Hz) activity.

Figures 6, 7 show the temporal evolution of the alpha (8–12 Hz) and beta (20–30 Hz) activity with respect to the baseline (−0.4 to −0.2 s) level. In Figure 6, the alpha topography is plotted with a 0.2 s interval for the left and right index finger stimulation conditions (two upper rows), along with the difference between conditions (bottom row). From the first 200 ms interval after the stimulus onset, a clear contralateral suppression in the alpha band was observed in sensors overlying the SMI regions. The contralateral alpha suppression became stronger between 0.2 and 0.6 s and showed a reduction in strength during the interval between 0.6 and 1.0 s. The difference in the alpha power between the left and right stimulation conditions was most prominent between 0.2 and 0.6 s after onset, due to the strong lateralization effect of each stimulation condition. On the other hand, a slight increase in alpha power was observed bilaterally over the posterior sensors for both conditions while strong suppression was observed for contralateral SMI sensors.

In Figure 7, the beta band (20–30 Hz) also exhibited a clear suppression with a similar sensor topography as the alpha band. The contralateral beta suppression was observed during the 0–0.2 s period, showed its strongest effect in sensors over SMI regions during the 0.2–0.4 s period, and decreased during the 0.4–0.6 s period, both for left and right stimulation conditions. The strongest beta power difference between conditions was observed between 0.2 and 0.4 s. Meanwhile, beta activity increased above baseline levels during the interval from 0.4 to 1.0 s in the posterior sensors. The power enhancement in the beta band in posterior sensors was observed bilaterally for both conditions, but didn't show notable differences between conditions.

**Figure 8:**
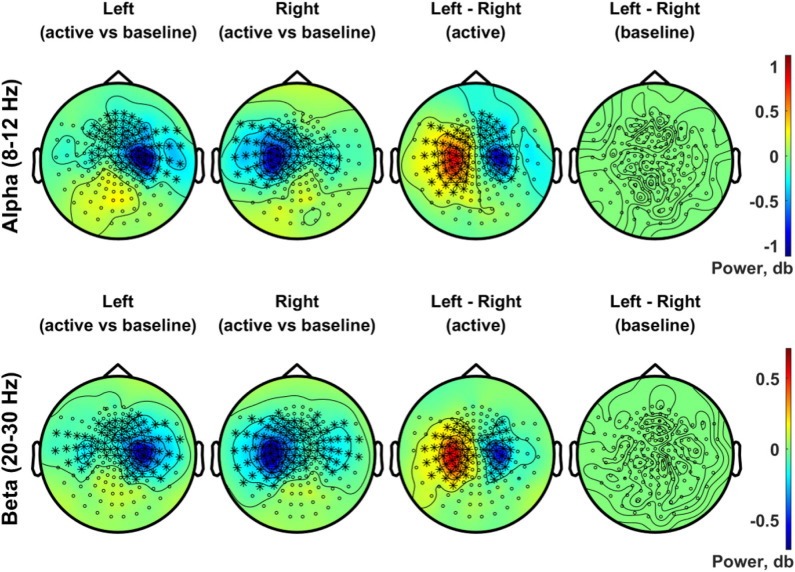
Topographic distribution of spectral power in the alpha band (8–12 Hz, upper panel) and in the beta band (20–30 Hz, lower panel). In the upper panel, the distributions of the alpha (8–12 Hz) power changes during an active period between 0.2 and 0.4 s after stimulus relative to the baseline period (−0.4 to −0.2 s) are plotted for the left (first column) and right (second column) index finger stimulation conditions. For each stimulation condition, statistical significance of the power change relative to baseline was evaluated by using a cluster-based permutation test (*N* = 29, 1,000 permutations, cluster-defining threshold *p* < 0.05, α = 0.05). In addition, the difference in the alpha power for the active period between left and right stimulation conditions, as well as the difference in baseline alpha activity between conditions, was also statistically tested for significance (*N* = 29, cluster-based permutation test, 1,000 runs, cluster-defining threshold *p* < 0.05, α = 0.05). Channels associated with significant clusters are indicated by asterisks. In the lower panel, the same analyses were applied as the upper panel but for oscillatory powers in the beta band (20–30 Hz). All plots are obtained for planar gradiometers.

Figure 8 presents the scalp topography of oscillatory power changes in the alpha band (top row, 8–12 Hz) and in the beta band (bottom row, 20–30 Hz) for an active period between 0.2 and 0.4 s in the left and right stimulation conditions, respectively, relative to baseline levels. Alpha power in the active period was significantly suppressed in the hemisphere contralateral to the stimulated side for each condition (1,000 permutations, *p* < 0.05 with a paired *t*-test for each sample and a significance level of 0.05). The statistical comparison of the left and the right index finger stimulation conditions clearly indicates the lateralization of alpha power in SMI sensors. The beta power suppression, on the other hand, was significant in bilateral SMI regions, showing considerable overlap in contralateral SMI sensors associated with the alpha power suppression. A significant difference in beta modulations between conditions was observed with a group of similar sensors as the alpha modulations. There was no significant difference in baseline activity of alpha and beta band oscillations between the two stimulation conditions.

**Figure 9:**
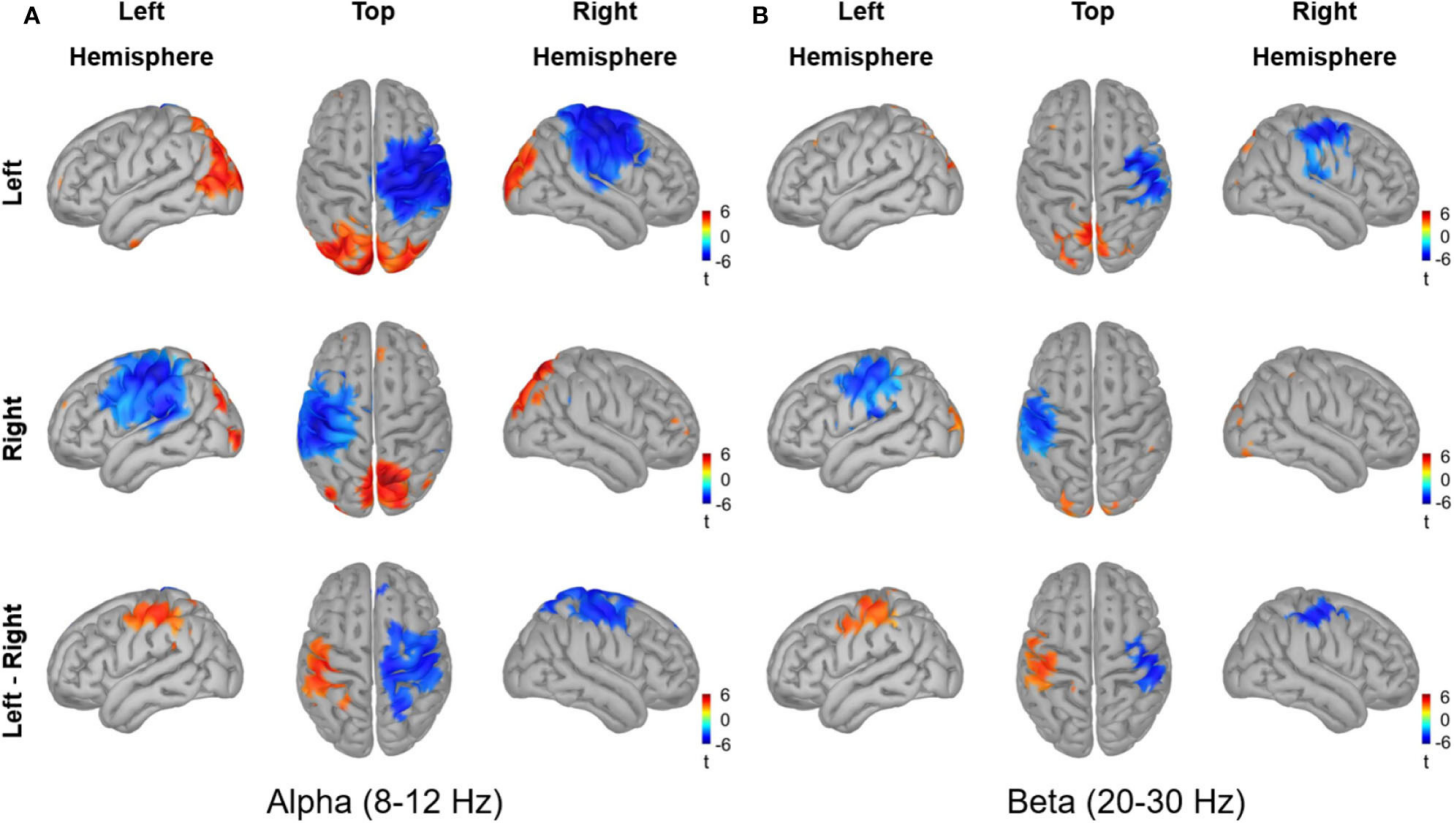
Source reconstruction of the oscillatory activities in the alpha band (8–12 Hz) and in the beta band (20–30 Hz) induced by vibrotactile stimulation. The grand averaged oscillatory power changes **(A)** in the alpha band (8–12 Hz) and **(B)** in the beta band (20–30 Hz) are presented in simultaneous views from the left hemisphere, top, and the hemisphere. The dB transformed spectral power relative to the baseline (−0.4 to −0.2 s) was averaged over an active period between 0.2 and 0.4 s after stimulus onset, and then statistically tested for the significance using a permutation paired *t*-test (10,000 randomizations, α = 0.01, FDR corrected). The statistical t-maps of areas showing significant power change in the active period relative to the baseline are plotted for the left index finger stimulation condition (top panel) and for the right index finger stimulation condition (middle panel), respectively. In the lower panel, the statistical t-maps of areas showing significant differences between left and right stimulation conditions are plotted, with the blue color indicating lower activity for the left index finger stimulation compared with the right index finger stimulation.

The source reconstruction for the alpha and beta activity during the active period (0.2–0.4 s) relative to baseline is shown in Figure 9. Vitrotactile stimulation of either index finger significantly suppressed alpha activity over a wide cortical region in and around the contralateral SMI and SII cortices, whereas alpha activity in the bilateral posterior regions was significantly enhanced (10,000 randomizations, α = 0.01, FDR corrected). The statistical comparison between left and right stimulations revealed significant difference in the SMI areas from both hemispheres, involving a broader region in the right hemisphere. The beta power change relative to the baseline level, on the other hand, was significant over a narrower region comprising the SMI and SII cortices contralateral to the side of vibrotactile stimulation. Significant difference in beta power modulations between stimulation conditions was observed in the SMI cortex of each hemisphere. The SII and the posterior regions did not show any significant difference between conditions for both alpha and beta activity.

## 4. Discussion

In the present work, we investigated the evoked and induced MEG responses to high frequency tactile vibrations at 150 Hz, using an MEG-compatible polymer based stimulation device. The somatosensory M50 and M100 peak responses were observed in the evoked magnetic fields following vibrotactile stimulation of the left and right index fingers with source activity localized in and around the primary sensorimotor (SMI) areas contralateral to the stimulated side. Time-frequency analysis revealed that both alpha (8–12 Hz) and beta (20–30 Hz) oscillations during the mid-latency period (0.2–0.4 s) were suppressed in the SMI and SII regions with contralateral dominance, while both oscillations were enhanced in posterior regions.

The time-lock analysis revealed prominent peak responses at 56 ms (M50) and at 100 ms (M100) for both stimulation conditions. The M50 was the first peak of the gERF in the present study, showing clear dipolar field distributions in the contralateral side (Figure 3). The source activation for the M50 was primarily observed in contralateral SMI cortices for each stimulation condition with strongest *z* values in the hand representation areas (Figure 4A). The M50 has been reported as an initial response to vibrotactile stimulation in previous studies (Hashimoto et al., 1998, 1999; Jousmäki and Hari, 1999; Iguchi et al., 2005), elicited in the primary somatosensory cortex contralateral to the stimulated side with latencies at about 40–60 ms. The difference in cortical responses between left and right index finger stimulation conditions was significant in large regions over the SMI cortex of both hemispheres, presumably due to the spatial spreading of the source estimate for the strong M50 sources.

The M100 response, on the other hand, was not as clearly observed as the M50 response in the field distributions (Figure 3), while the bilateral responses were visually identifiable. The source activation for the M100 was observed over the SMI and SII areas contralateral to the stimulation side, with no discernible ipsilateral response (Figure 4B). Due to weak cortical activations for M100, significant differences between conditions were predominantly observed in the left SMI area, driven by a stronger contralateral response to right index finger stimulation. The somatosensory M100 is considered to be the peak response observed between 70 and 140 ms after somatosensory inputs, originating predominantly from bilateral SII cortices (Hari et al., 1993; Mauguiere et al., 1997; Jousmäki and Hari, 1999; Hoechstetter et al., 2001) with larger responses in the contralateral than in the ipsilateral hemisphere. The sensitivity of M100, however, depends on experimental and psychological factors including stimulus intensity (Jousmäki and Forss, 1998; Jones et al., 2007), interstimulus interval (ISI) (Hari et al., 1993; Wikström et al., 1996; Karhu and Tesche, 1999; Mertens and Lütkenhöner, 2000; Hamada et al., 2002), attention (Mauguiere et al., 1997; Mima et al., 1998; Karhu and Tesche, 1999; Hoechstetter et al., 2000; Iguchi et al., 2005), and habituation (Inoue et al., 2002). The M100 response also shows considerable inter-individual variability in peak latencies, source locations, and dipole orientations (Hari et al., 1993; Hashimoto et al., 1998). We speculated that the effective ISI of 2 s, successive monotonous stimuli for a long recording time (10 min per session), and the relatively low vibration intensity used in our experiments, together with individual variability, might have reduced the M100 response in bilateral SII areas. The limitations in the source model due to the lack of individual MRIs might also have contributed to make the detection of SII activity difficult in our study, considering the small and complicated folding structure of SII areas (Hari et al., 1993). It is notable, however, that earlier neuromagnetic studies on vibrotactile stimulation reported the source of the M100 to be in the contralateral SI (Hashimoto et al., 1998). In order to interpret our results for the M100 response, whether it is due to the reduced sensitivity or whether it is caused by specific neural mechanisms associated with vibrotactile perception needs to be further investigated with optimized experimental setups and individual MRIs in future research.

While evoked responses are time-locked averaged signals elicited by stimulation events, induced responses represent modulations of neuronal oscillations caused by the stimulation (Pfurtscheller and Da Silva, 1999; Tallon-Baudry and Bertrand, 1999; David et al., 2006), and are frequently measured in the form of event-related synchronization (ERS) or desynchronization (ERD), implying a spectral power increase or decrease, respectively, relative to the baseline level (Pfurtscheller and Da Silva, 1999; Neuper et al., 2006). The induced responses in Figure 5 displayed desynchronization both at ~12 Hz between 100 and 800 ms and at ~22 Hz between 100 and 400 ms, and synchronization at ~18 Hz between 600 and 800 ms. These ERD/ERS effects were predominant in channels located in the hemisphere contralateral to stimulated side. Our results are consistent with previous findings on tactile stimulations (Cheyne et al., 2003; Bauer et al., 2006; Andersen and Lundqvist, 2019), and suggest a similar neuronal processing for tactile signals transmitted by different types of mechanoreceptors. Only ERD effects showed a significant difference between conditions, and we investigated the modulations in alpha (8–12 Hz) and beta (20–30 Hz) band activity in more detail.

Vibrotactile stimulation of the left index finger resulted in a significant suppression of alpha band activity in sensors overlying the right SMI area, and vice versa, as shown in Figure 8. The alpha suppressions were localized to widely distributed cortical areas including SMI and SII contralateral to the stimulation side, as shown in Figure 9A. An unexpected finding in the present study was that alpha activity was enhanced in the posterior regions bilaterally, whereas it was selectively suppressed in the SMI and SII regions. A reduction in alpha power is considered to indicate activated brain regions for sensory information processing (Pfurtscheller and Da Silva, 1999; Singh et al., 2002; Neuper et al., 2006). Meanwhile, previous studies have shown that alpha power increases to inhibit task-irrelevant regions while it decreases to facilitate processing in task-relevant regions (Pfurtscheller et al., 1996; Neuper et al., 2006; Jensen and Mazaheri, 2010; Haegens et al., 2012). Our data can be interpreted as suggesting that the contralateral SMI and SII areas were activated to process vibrotactile stimuli and that the bilateral posterior areas were functionally inhibited for optimal task performance. Interestingly, a significant difference in the alpha suppression effect between conditions was observed in more broadly distributed areas in the right hemisphere, mainly driven by alpha suppression in response to left index finger stimulation. Our result supports previous observations that more broadly distributed cortical areas are activated for tactile perception of the subdominant hand and that a more efficient and cortically concentrated neural process is developed for tactile perception of the dominant hand (Pihko et al., 2009; Yang et al., 2018). On the other hand, no significant difference was found during left or right index finger stimulations in bilateral enhancements of alpha activity in the posterior areas.

The beta band activity was also suppressed by the vibrotactile stimuli, as shown in Figure 8, exhibiting more bilateral distributions of significant suppression effects in sensors over the SMI areas with a strong contralateral bias. Source analysis, however, localized significant beta suppression effect to the finger representation area of the SMI and SII cortices in the contralateral side only (Figure 9B). Earlier studies have reported that tactile stimulation induced a suppression of beta activity in the contralateral sensorimotor cortex (Cheyne et al., 2003; Bauer et al., 2006; Gaetz and Cheyne, 2006). Sensorimotor beta activity is considered to reflect motor-cortex excitability (Parkkonen et al., 2015), with suppression and rebound representing activation and deactivation of the motor cortex, respectively. Beta oscillations are also suggested to play important roles in top-down processing, long-range communication, and preservation of the current brain state (Spitzer and Haegens, 2017).

In fact, previous studies have shown that electrical somatosensory stimulation induces alpha and beta ERD over the bilateral SI with a contralateral predominance (Hirata et al., 2002; Penna et al., 2004). In the present study, however, neither alpha nor beta suppression was significant in the ipsilateral sensorimotor area, although bilateral suppressions in both alpha and beta band activity were presumed based on the sensor space analyses. On the other hand, our results indicate that both alpha and beta activity are suppressed in the contralateral SII areas after vibrotactile stimulation. Beta suppression has been reported bilaterally in secondary somatosensory cortex after median nerve stimulation (Penna et al., 2004). The secondary somatosensory cortices are considered to perform higher order cognitive functions, such as sensorimotor integration (Huttunen et al., 1996; Inoue et al., 2002), integration of bilateral stimulations (Hoechstetter et al., 2001), attention (Mima et al., 1998; Chen et al., 2008), and stimulus discrimination (Iguchi et al., 2007). Although limitations of our data, including low signal-to-noise ratio (SNR) and low spatial resolution due to the lack of individual MRIs, prevented us from precisely determining the temporal dynamics in SMI and SII regions, our data suggest that alpha and beta activity in both SMI and SII regions are closely involved in the processing of vibrotactile information with contralateral dominance. Further studies are needed to understand the temporal dynamics of alpha and beta oscillations in SMI and SII as well as the interaction between SMI and SII during vibrotactile information processing. To achieve reliable results, we need to improve the SNR of the signals by using both advanced design of the stimulation device for stronger intensity and more appropriate experimental paradigms for high vigilance levels of participants.

Previous MEG studies have shown that the somatosensory information processing involves activation in primary and secondary somatosensory cortices (Forss et al., 1994; Hari and Forss, 1999; Karhu and Tesche, 1999; Kakigi et al., 2000; Hoechstetter et al., 2001). Most studies, however, used electrical stimulation of peripheral nerves to activate a broad range of cutaneous receptors or tactile stimulation to activate mainly slowly adapting mechanoreceptors (Forss et al., 1994; Rossini et al., 1996; Jousmäki, 2000; Inoue et al., 2005; Hautasaari et al., 2019). Much less is known about brain responses activated by rapidly adapting mechanoreceptors, i.e., Meissner and Pacinian corpuscles. The threshold for detecting high frequency vibrations above 50 Hz is known to be determined by Pacinian corpuscles (Gescheider et al., 2002), with its greatest sensitivity in the range of 100–300 Hz (Griffin, 2012).

We believe that our study is the first report of oscillatory neuronal activity induced by activating the rapidly adapting mechanoreceptors, presented together with evoked responses. Somatosensory evoked responses have been extensively used for developmental studies in human somatosensory systems (Pihko et al., 2009; Nevalainen et al., 2014; Whitehead et al., 2019). Impaired somatosensory processing is associated with various neurodevelopmental disorders, such as autism spectrum disorders (ASD), attention deficit hyperactivity disorder (ADHD), and cerebral palsy (CP) (Cascio, 2010), and strong evidences suggests that somatosensory cortical oscillations, as well as evoked responses, are aberrant in children with developmental disorders (Dockstader et al., 2008; Marco et al., 2012; Papadelis et al., 2014; Pihko et al., 2014; Kurz et al., 2015; Gaetz et al., 2017). Our vibrotactile device may be used to design naturalistic and child friendly experiments to diagnose neurodevelopmental disorders in clinical applications. Moreover, understanding the neural mechanisms underlying vibrotactile perception is crucial for improving the cognitive performance of vibrotactile feedback systems in rehabilitation, navigation, virtual environment, and teleoperations, etc. (Jones and Sarter, 2008; Alahakone and Senanayake, 2009; Choi and Kuchenbecker, 2013; Culbertson et al., 2018). While further experiments and analyses are required to elucidate details of the cortical processes associated with vibrotactile perception, our results may contribute toward the understanding of neuronal responses to selective activation of rapidly adapting mechanoreceptors, thereby giving insights into the neural basis for subjective evaluation of vibrotactile signals.

## Data Availability Statement

The raw data supporting the conclusions of this article will be made available by the authors, without undue reservation.

## Ethics Statement

The studies involving human participants were reviewed and approved by Institutional Review Board on Human Subjects Research and Ethics Committee of Korea Research Institute of Standards and Science (KRISS). The patients/participants provided their written informed consent to participate in this study.

## Author Contributions

HK integrated the vibrotactile stimulator into the existing MEG system. M-YK recruited the participants, performed the MEG experiments, analyzed the data, prepared the figures, and wrote the manuscript. All authors conceptualized the study, designed the experiments, discussed the results, revised the manuscript, and approved the final version of the manuscript for submission.

## Conflict of Interest

The authors declare that the research was conducted in the absence of any commercial or financial relationships that could be construed as a potential conflict of interest.
