# Supplementary material for: Vibration Alert to the Brain: Evoked and Induced MEG Responses to High-Frequency Vibrotactile Stimuli on the Index Finger of Dominant and Non-dominant Hand
Source: Front Hum Neurosci. 2020 Nov 5;14:576082. doi: 10.3389/fnhum.2020.576082 (PMC7674801; doi:10.3389/fnhum.2020.576082)
Supplement: Supplementary file 1 [file Data_Sheet_1.pdf]

Supplementary Material

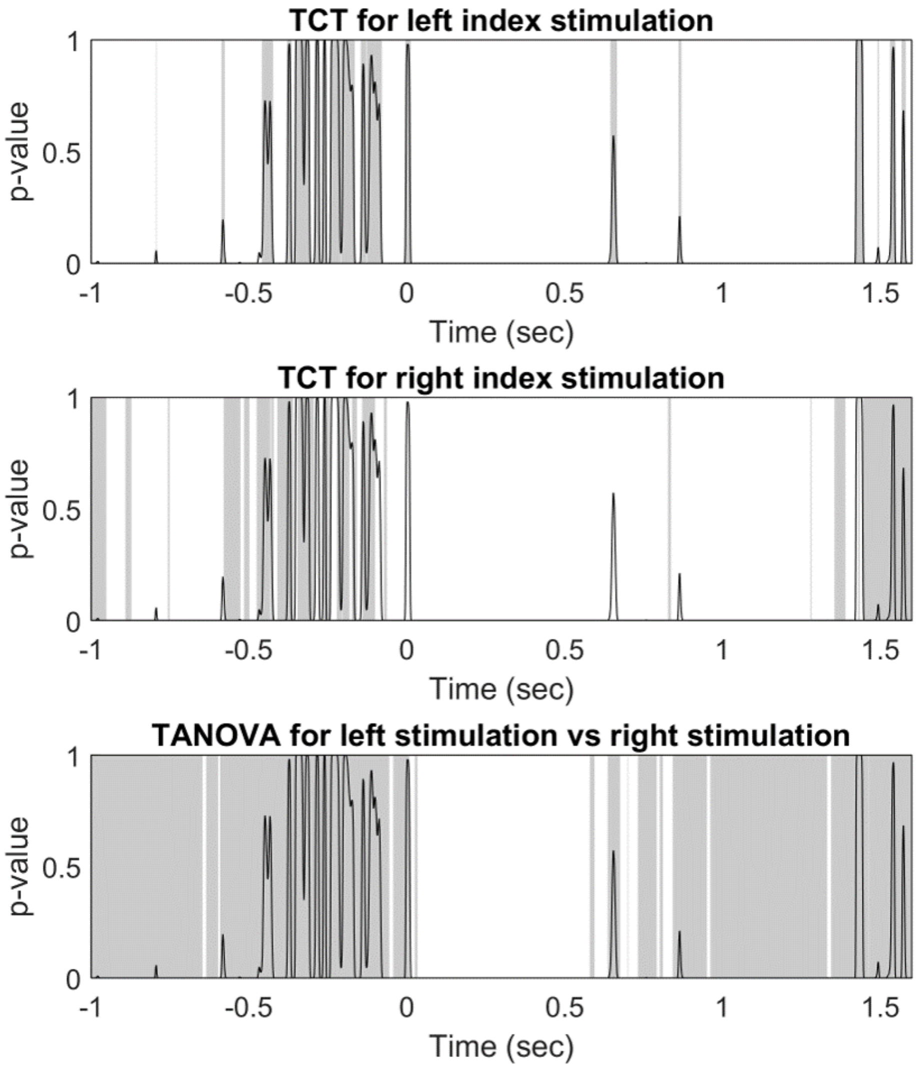

Figure S1. Results of topographic statistical analyses. The p-value of a topographic consistency test (TCT) applied to the grand averaged event related fields (gERFs) obtained from the left index finger stimulation (top) and from the right index finger stimulation (middle) are shown (5000 randomizations, p=0.05). The p-value of topographic ANOVA (TANOVA) for the comparison between gERFs of each condition is displayed in the bottom panel (5000 randomizations, p=0.05). Gray areas mark non-significant time points while white areas mark significant time points.

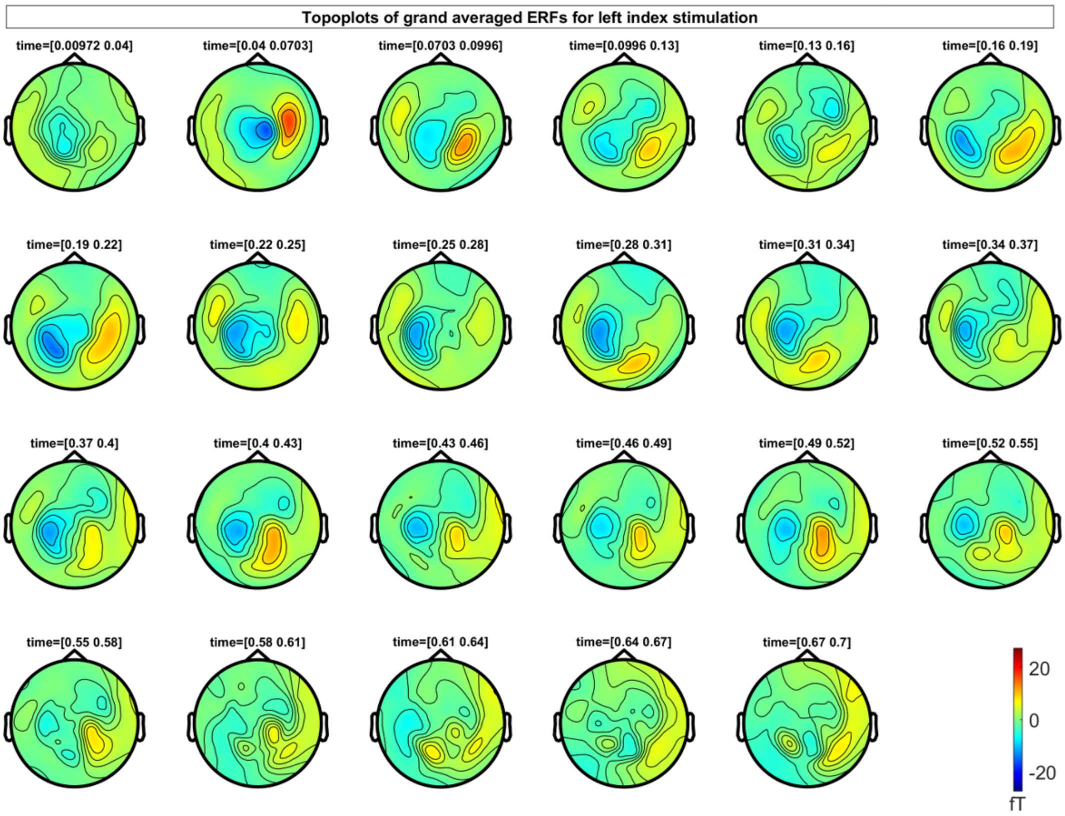

Figure S2. Temporal development of topographical distributions of event related fields (ERFs) in response to left finger index stimulation. The temporal development of topographical patterns is visualized by using the mean values of the grand averaged ERFs for a 0.03 s time window advanced from 0.01 s to 0.7 s in steps of 0.03 s without overlapping. All plots are obtained for axial gradiometers.

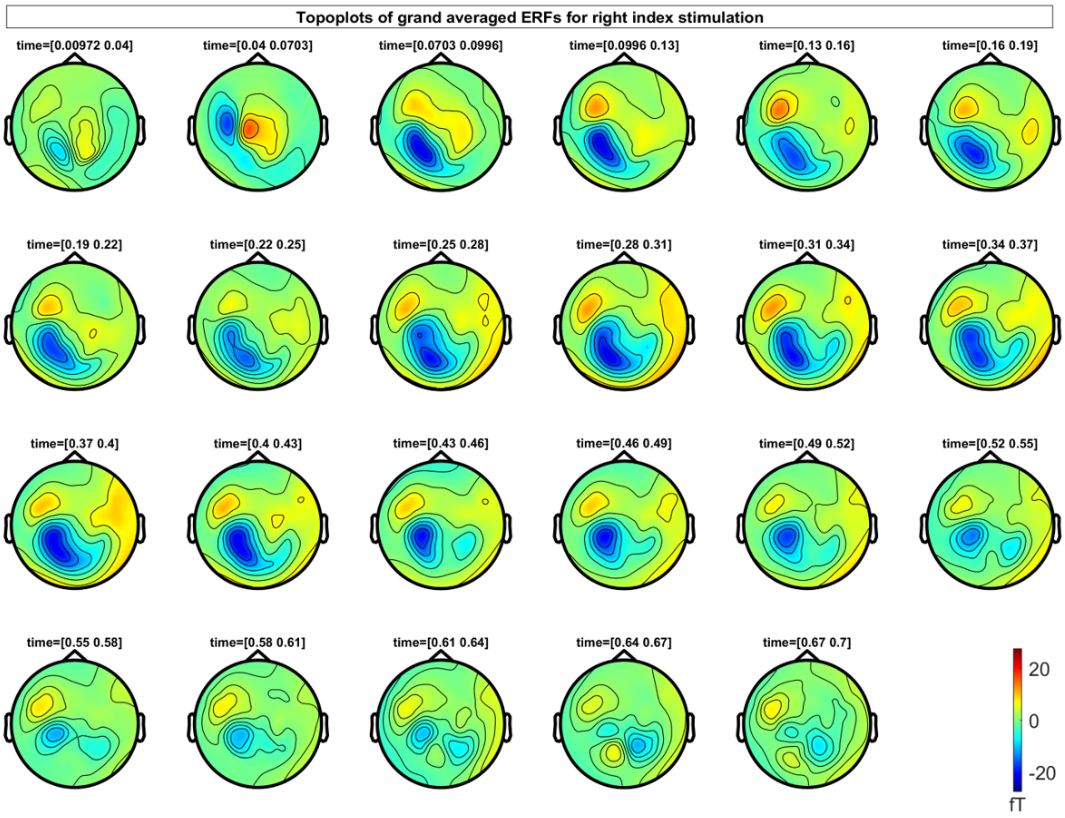

Figure S3. Temporal development of topographical distributions of event related fields (ERFs) in response to right index finger stimulation. The temporal development of topographical patterns is visualized by using the mean values of the grand averaged ERFs for a 0.03 s time window advanced from 0.01 s to 0.7 s in steps of 0.03 s without overlapping. All plots are obtained for axial gradiometers.

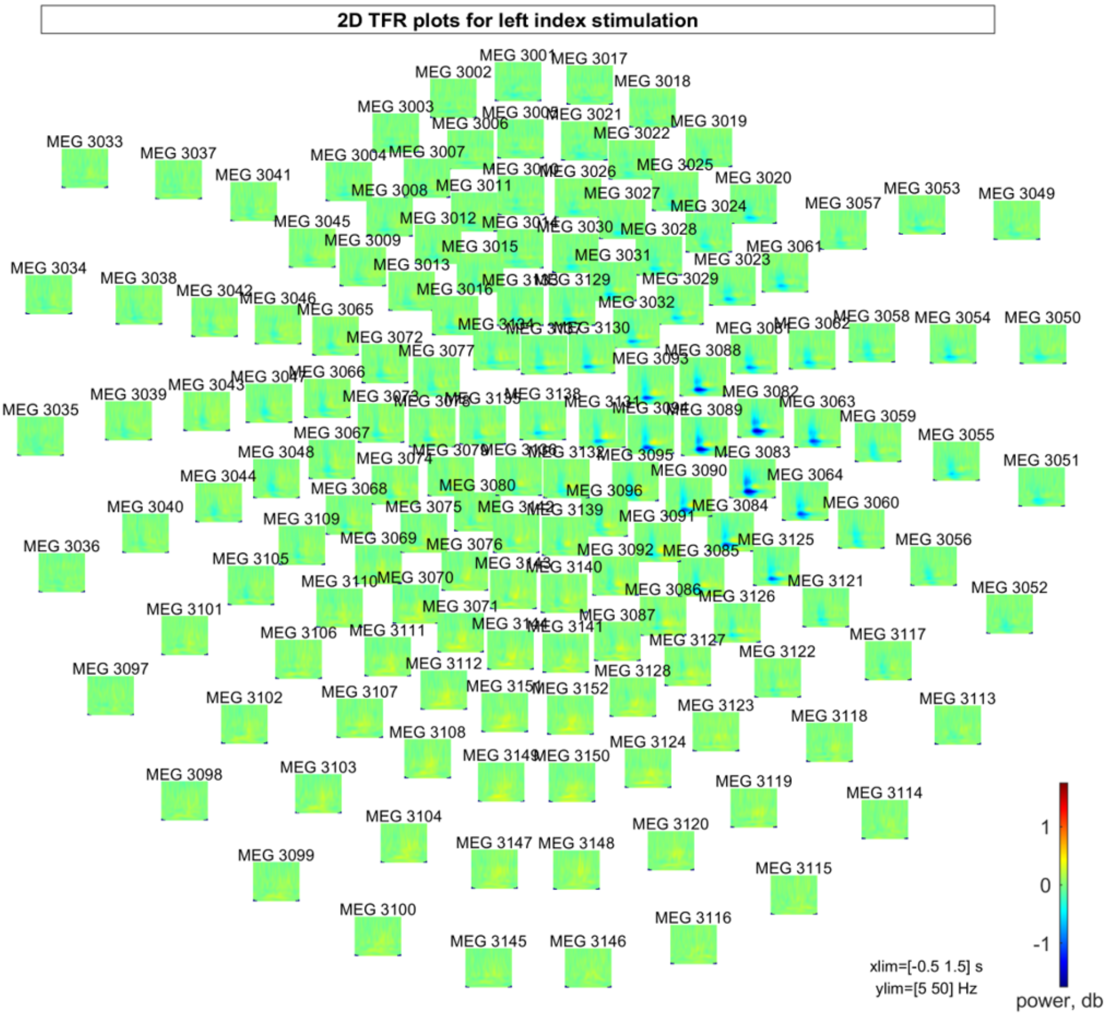

Figure S4. 2D plots of grand averaged spectral power changes relative to baseline induced by left index finger stimulation. The time-frequency representation (TFR) was obtained for a planar gradiometer configuration. The TFR power is shown from 5 to 50 Hz over the time period between -0.5 and 1.5 s. The red color indicates spectral power increase relative to baseline, whereas the blue color indicates spectral power decrease relative to baseline.

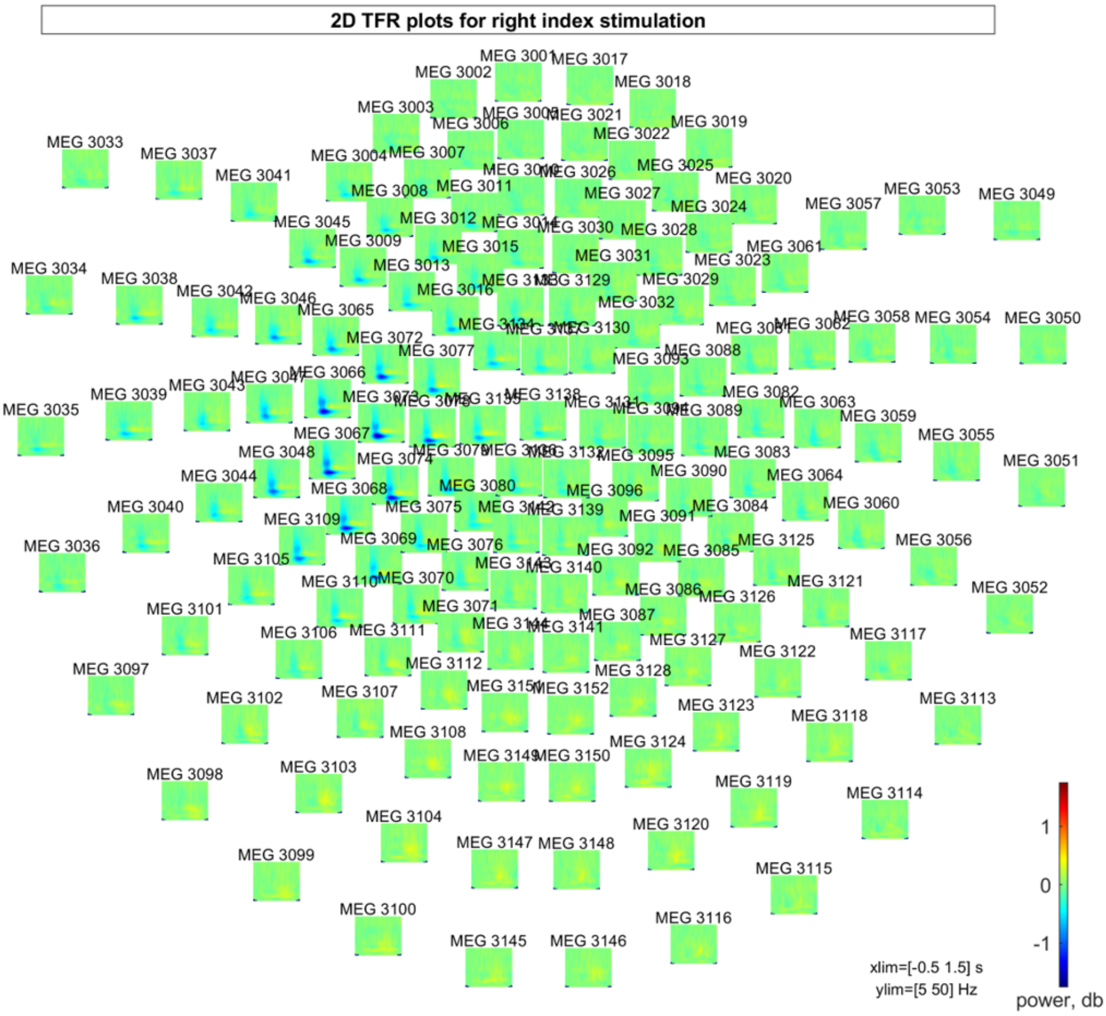

Figure S5. 2D plots of grand averaged spectral power changes relative to baseline induced by right index finger stimulation. The time-frequency representation (TFR) was obtained for a planar gradiometer configuration. The TFR power is shown from 5 to 50 Hz over the time period between -0.5 and 1.5 s. The red color indicates spectral power increase relative to baseline, whereas the blue color indicates spectral power decrease relative to baseline.

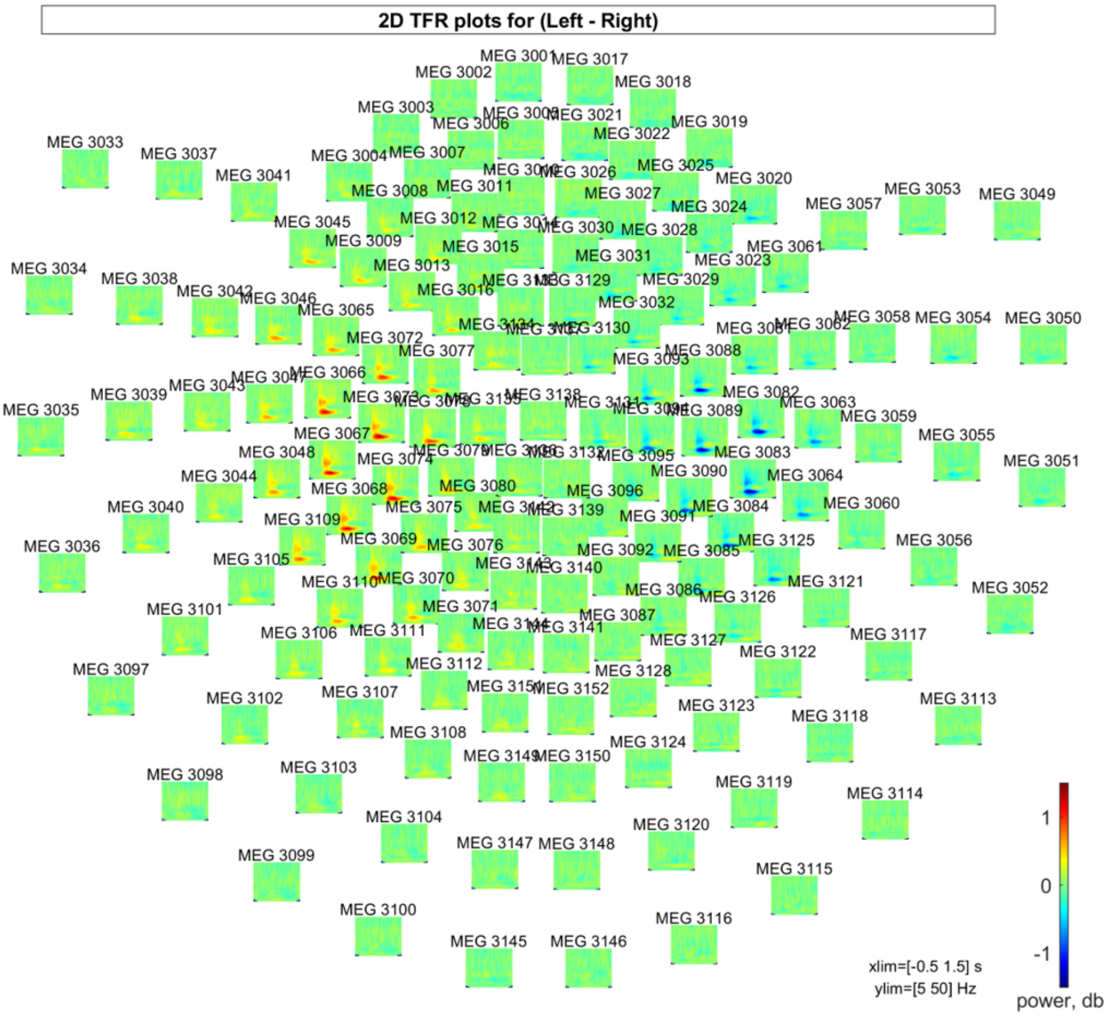

Figure S6. 2D plots of grand averaged spectral power differences between left and right index finger stimulation conditions. The time-frequency representation (TFR) was obtained for a planar gradiometer configuration. The TFR power difference is shown from 5 to 50 Hz over the time period between -0.5 and 1.5 s. The blue color indicates lower spectral activity for the left index finger stimulation compared with the right index finger stimulation, whereas the red color indicates lower spectral activity for the right index finger stimulation compared with the left index finger stimulation.

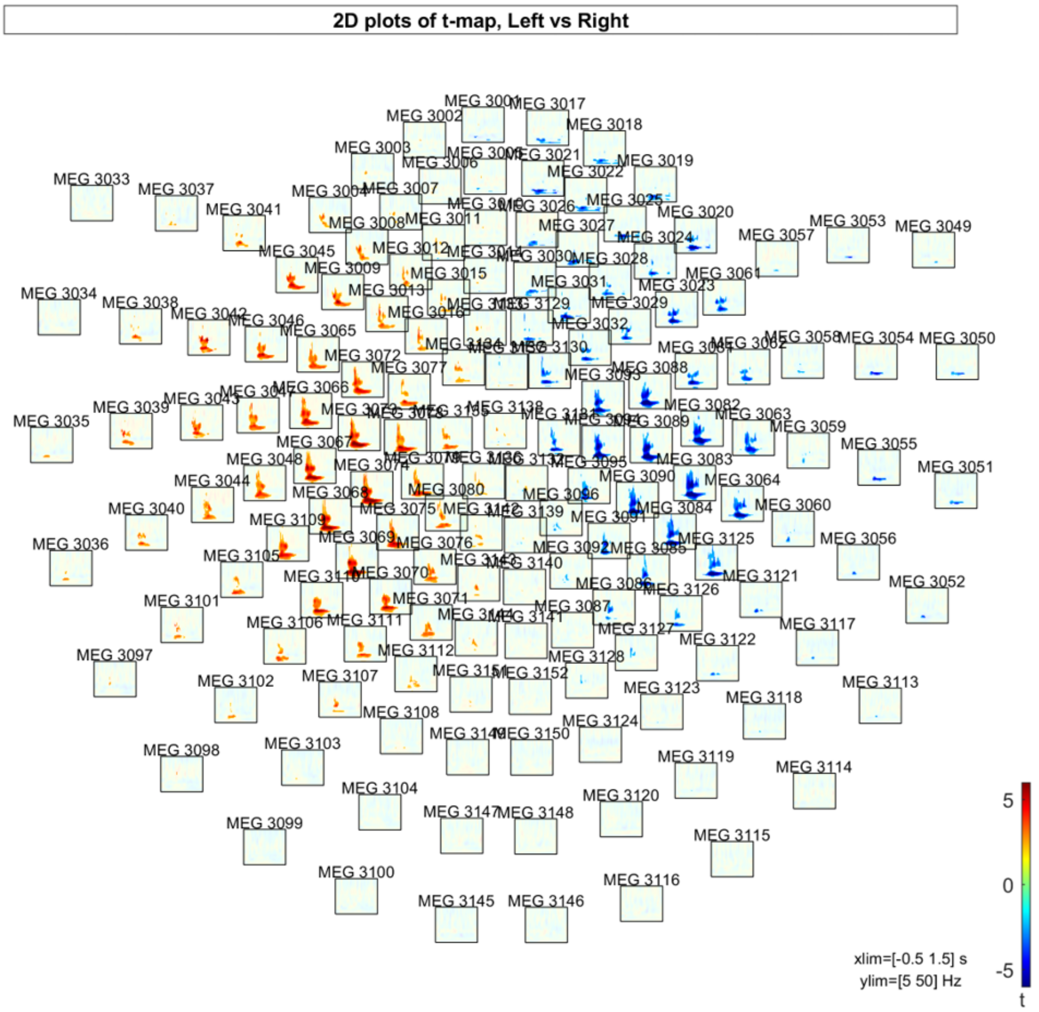

Figure S7. 2D plots of statistical t-maps for spectral power differences between left and right index finger stimulation conditions. The time-frequency t-map was obtained from 5 to 50 Hz over the time period between -0.5 and 1.5 s for a planar gradiometer configuration. Power differences associated with significant clusters are shown in non-blurred (N=29, a cluster-based permutation test, 1000 runs, a significance level of 0.05).
